# Bluetooth dataset for proximity detection in indoor environments collected with smartphones

**DOI:** 10.1016/j.dib.2024.110215

**Published:** 2024-02-19

**Authors:** Michele Girolami, Davide La Rosa, Paolo Barsocchi

**Affiliations:** Institute of Information Science and Technologies, National Research Council, (ISTI-CNR), Via G. Moruzzi, 1, 56124, Pisa, Italy

**Keywords:** CrowdSensing, Bluetooth, Indoor localization, Proximity, Cultural heritage

## Abstract

This paper describes a data collection experiment and the resulting dataset based on Bluetooth beacon messages collected in an indoor museum. The goal of this dataset is to study algorithms and techniques for proximity detection between people and points of interest (POI). To this purpose, we release the data we collected during 32 museum's visits, in which we vary the adopted smartphones and the visiting paths. The smartphone is used to collect Bluetooth beacons emitted by Bluetooth tags positioned nearby each POI. The visiting layout defines the order of visit of 10 artworks. The combination of different smartphones, the visiting paths and features of the indoor museum allow experiencing with realistic environmental conditions. The dataset comprises RSS (Received Signal Strength) values, timestamp and artwork identifiers, as long as a detailed ground truth, reporting the starting and ending time of each artwork's visit. The dataset is addressed to researchers and industrial players interested in further investigating how to automatically detect the location or the proximity between people and specific points of interest, by exploiting commercial technologies available with smartphone. The dataset is designed to speed up the prototyping process, by releasing an accurate ground truth annotation and details concerning the adopted hardware.

Specifications Table

**Table:** 

Subject	Computer science: Computer science application
Specific subject area	Indoor localization; proximity detection; internet of things;
Data format	Raw
Type of data	.csv files (dataset with Ground Truth annotation)
Data collection	Data are collected during the user's visits in a museum. Dataset is generated by 32 tests, 8 smartphones are used and 4 visiting path are tested. The 10 monitored artworks are equipped with a Bluetooth tag emitting Bluetooth's beacons at 2 Hz and −23 dBm. Collected data include timestamp, RSS value estimated by the receiving device, MAC address of the Bluetooth tag and UUID of the Bluetooth tag.
Data source location	Monumental Cemetery's museum, Piazza dei Miracoli, Pisa (IT) (GPS coordinates: 43.723958, 10.394980)
Data accessibility	Repository name: Mendeley Data Data identification number: 10.17632/sbhch9pxwh.1 Direct URL to data: https://data.mendeley.com/datasets/sbhch9pxwh/1
Related research article	M. Girolami, D. La Rosa, P. Barsocchi, A crowdsensing-based approach for proximity detection in indoor museums with Bluetooth tags, Ad Hoc Networks 154 (2024) 103367. doi: https://doi.org/10.1016/j.adhoc.2023.103367. URL https://www.sciencedirect.com/science/article/pii/S1570870523002871

## 1 Value of the Data

• This dataset provides information of the collected Bluetooth beacons during user's visits in an indoor museum. The collected data allow testing proximity detection algorithms based on the analysis of Bluetooth beacons (RSS analysis), at realistic conditions. Indeed, the dataset provides data captured with commercial smartphones and Bluetooth tags at real-world conditions.
• Researchers and start-up players can use this dataset in order to analyze and automatically detect proximity between subjects and point of interests at realistic conditions.
• The dataset can be used to also characterize how RSS (Received Signal Strength values vary across different devices and different conditions. Indeed, collected data also include non-proximity events, therefore it could be possible to investigate how RSS values vary while approaching to an artworks (a point of interest) or while leaving an artwork.
• The dataset presents also additional values: (i) a detailed Ground Truth of the proximity and non-proximity events, (ii) data are gathered with commercial devices, as a result the dataset provides non-biased traces of the RSS values and (iii) the experiments are designed to consider heterogeneous condition of the users involved

## 2 Background

The motivation behind this dataset is to provide useful data to test and reproduce proximity detection and indoor localization algorithms based on the analysis of Bluetooth RSS values, in an indoor environment [1], [2], [3], [4], [5]. Traditional approaches to detect proximity between visitors and artworks include the use of camera, self-reported questionnaires or diaries compiled by an observers watching at scene. Such approaches, although useful, do not automatically provide the exact starting and ending time of a museum visit as can be obtained with the use of Bluetooth tags.

The data collection has been designed to reproduce realistic conditions during the museum's visits. Visitors act in a natural way and external people has access to the museum at the same time. Data are collected with commercial devices, and we vary the adopted devices’ models [6,7].

This article is related to a research article reported in Girolami et al. [1] in which we analyze the performance of some proximity detection algorithms. The preset dataset allows to: reproduce the obtained results, design, test and evaluate new strategies for proximity detection and indoor localization in indoor museums.

## 3 Data Description

This dataset provides a set of CSV (comma separated values) files containing information associated with Bluetooth beacons recorded by smartphones. The dataset is organized in 32 runs and it is conducted in an indoor museum. We refer to each run as *R_i_*, each run is executed with a specific smartphone model and with a specific visiting layout L_j_. In particular, we adopted the following smartphone models:

• Honor 9
• Honor 8(1)
• Honor 8(2)
• Honor 9
• Huawei P8
• Huawei P30
• Pixel 4a
• Redmi 8

Concerning the visiting layouts, they are denoted as: L_1_, L_2_, L_3_, L_4_. Each layout defines a specific visit order of the artworks exhibited in the museum.

We report the following tables from which it is possible to quickly extract:

• the list of runs executed with each smartphone, as reported in Table 1;

**Table 1:** List of runs executed with a specific smartphone.

Smartphone	Run ID
Honor 9	R4, R12, R20, R28
Honor 8–1	R6, R14, R22, R30
Honor 8 PRO	R7, R15, R23, R31
Honor 8–2	R3, R11, R19, R27
Huawei P8	R8, R16, R24, R32
Huawei P30	R5, R13, R21, R29
Pixel 4a	R1, R9, R17, R25
Redmi 8	R2, R10, R18, R26
• the list of runs executed according to each of the 4 visiting layouts, as reported in Table 2.

**Table 2:** List of runs executed according to a specific visiting layout. We also report the artwork's order for each visiting layout.

Visiting layout	Artwork's order	Run ID
L_1_	1,2,3,4,5,6,7,8,9,10	R1, R2, R3, R4, R5, R6, R7, R8
L_2_	1,3,4,7,9,8,2,5,6,10	R9, R10, R11, R12, R13, R14, R15, R16
L_3_	10,5,6,3,2,8,9,7,1,4	R17, R18, R19, R20, R21, R22, R23, R24
L_4_	6,8,9,5,7,3,2,4,1,10	R25, R26, R27, R28, R29, R30, R31, R32

Files in the dataset are organized in folders, each folder is named with the corresponding test name, e.g. Rj and inside each folder, we release two files according to the following naming convention:

• <timestamp>-beacons.csv: this log contains the raw data, whose file format is reported below;
• <timestamp>-proximity.csv: this log contains the Ground Truth of the proximity and non-proximity events associated with the <timestamp>-beacons.csv file. In particular, the file reports the starting time and the ending time of the artwork's visits.

The file format of Bluetooth beacons is the following:

• timestamp: the timestamp of reception of the Bluetooth beacons;
• name: the name of the received beacons;
• address: the MAC address of the received beacons;
• major: the artwork's identifier;
• RSSI: the Received Signal Strength Indicator in dBm unit estimated by the smartphone and associated with the Bluetooth beacon.

Concerning the Ground Truth, the file format is the following:

• timestamp: the timestamp of reception of the Bluetooth beacons;
• event: the name of the two relevant events: check-in when a visitor is in proximity with a specific location, check-out when a visitor move away from the location;
• location: the artwork's identifier, matching with the major column of the <timestamp>-beacons.csv file.

## 4 Experimental Design, Materials and Methods

### 4.1 Design

The collected dataset has been designed to reproduce proximity and non-proximity events in an indoor museum under realistic conditions. To this purpose, the requirements we consider in preparation for the data collection campaign can be summarized as follows:

• natural behavior: Visitors are free to move in indoor environment. They can walk, rest and visit the exhibited artworks acting in a natural way;
• device heterogeneity: smartphones used for the data collection are commercial devices, equipped with the Bluetooth radio interface. We test 8 different commercial models;
• multiple visiting layouts: we define 4 different orders of visits, each comprising 10 artworks;
• realistic museum's conditions: tests were conducted during opening days, other visitors were present, including student's groups and tourist guides.

**Fig. 1:**
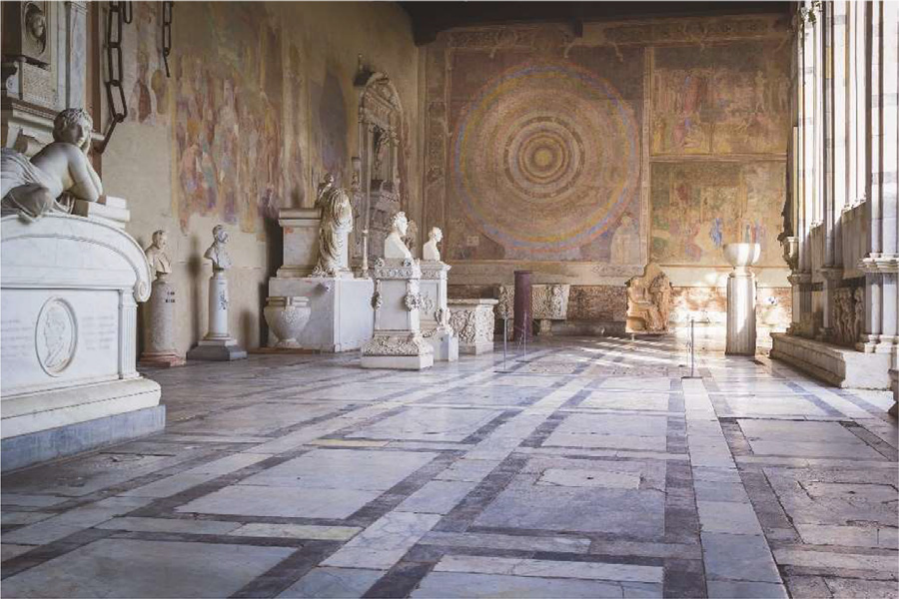
Example of artwork's layout in the Monumental Cemetery's museum of Pisa.

Tests have been conducted by the Monumental Cemetery's museum in Piazza dei Miracoli, Pisa (IT). The museum is organized with long and wide corridors, and on the left and right sides, artworks are positioned. Corridors are characterized by semi-opened columns on one side while, on the other side, frescos are present. Historical tombstones are also present, tiling the whole floor, as shown in Fig. 1.

For the purpose of our tests, we monitor 10 artworks, relatively close to each other. Distances between artworks can vary from 1.5 m to 5 m.

### 4.2 Material

**Fig. 2:**
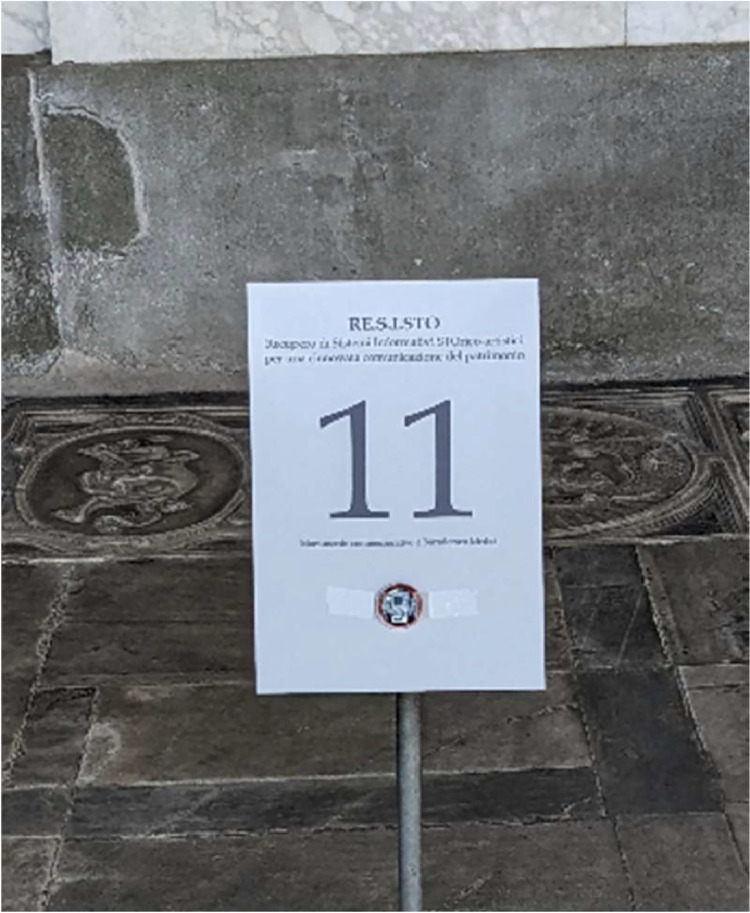
Installation of the Bluetooth tag.

The material we used for collecting the dataset is composed by Bluetooth tags applied to the artworks. Tags are produced by GlobalTag, they can advertise different types of Bluetooth beacons, such as iBeacons and EddyStone. We configure the tag to emit iBeacon messages at 2 Hz advertisement frequency and −23 dBm as emission power. Tags are applied to artworks without the use of any damaging material (paste, tape, screw etc.). More specifically, each artwork is equipped with an information sign and we applied the Bluetooth tag to the sign, as shown in Fig. 2.

Concerning the receiving device, we develop a mobile application for Android OS that is able to listen and log Bluetooth beacons. The mobile application is named R-app [5] and is based on the cross-platform React Native framework. Its purpose is to autonomously identify the proximity of visitors to various points of interest within a specific region, such as artworks within an indoor museum.

### 4.3 Methodology

**Fig. 3:**
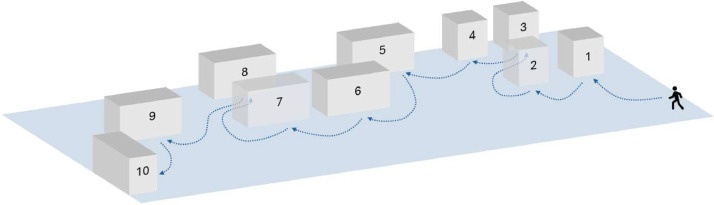
Example of visiting layout. The visitor follows the reported path, resting in front of each artwork for 2 min, approximately.

Concerning the adopted methodology, visitors follow different visiting layouts. Visitors walk at a regular pace, they rest in front of each artwork for 2 min, and then they walk to the next artwork, according to the visiting layout. We report in Fig. 3 an example of visiting layout. The user follows the blue line, resting in front of each artwork for 2 min. As reported in the figure, some artworks are close to each other (e.g. artworks with ID 1, 2, 3 and 4).

## Limitations

Experiments reported in this work can be improved following two possible lines of extension. On the one hand, data collection could be achieved during different months of the year (spring, summer and winter time) so that to also highlight any possible variation in terms of number of visitors and their impact on the RSS values estimated by the smartphones. On the other hand, we consider interesting to extend the number of monitored artworks and to increase the visiting layouts.

## Ethics Statement

Authors have read and follow the ethical requirements for publication in Data in Brief and confirming that the current work does not involve human subjects, animal experiments, or any data collected from social media platforms.

## CRediT authorship contribution statement

**Michele Girolami:** Conceptualization, Methodology, Supervision, Investigation, Visualization. **Davide La Rosa:** Conceptualization, Methodology, Investigation, Visualization. **Paolo Barsocchi:** Conceptualization, Methodology, Supervision, Investigation.

## Data Availability

A Bluetooth Dataset for Proximity Detection in Indoor Environments Collected with Smartphones (Original data) (Mendeley Data).
